# Prognostic Value of Echocardiographic Right Ventricular Function Parameters in the Presence of Severe Tricuspid Regurgitation

**DOI:** 10.3390/jcm10112266

**Published:** 2021-05-24

**Authors:** Matthias Schneider, Varius Dannenberg, Andreas König, Welf Geller, Thomas Binder, Christian Hengstenberg, Georg Goliasch

**Affiliations:** Department of Internal Medicine II, Medical University of Vienna, A-1090 Vienna, Austria; varius.dannenberg@meduniwien.ac.at (V.D.); andreas.koenig@sono4you.org (A.K.); n1629136@students.meduniwien.ac.at (W.G.); thomas.binder@meduniwien.ac.at (T.B.); christian.hengstenberg@meduniwien.ac.at (C.H.); georg.goliasch@meduniwien.ac.at (G.G.)

**Keywords:** TAPSE, FAC, GLS, RVF, TR

## Abstract

Background: Presence of severe tricuspid regurgitation (TR) has a significant impact on assessment of right ventricular function (RVF) in transthoracic echocardiography (TTE). High trans-valvular pendulous volume leads to backward-unloading of the right ventricle. Consequently, established cut-offs for normal systolic performance may overestimate true systolic RVF. Methods: A retrospective analysis was performed entailing all patients who underwent TTE at our institution between 1 January 2013 and 31 December 2016. Only patients with normal left ventricular systolic function and with no other valvular lesion were included. All recorded loops were re-read by one experienced examiner. Patients without severe TR (defined as vena contracta width ≥7 mm) were excluded. All-cause 2-year mortality was chosen as the end-point. The prognostic value of several RVF parameters was tested. Results: The final cohort consisted of 220 patients, 88/220 (40%) were male. Median age was 69 years (IQR 52–79), all-cause two-year mortality was 29%, median TAPSE was 19 mm (15–22) and median FAC was 42% (30–52). In multivariate analysis, TAPSE with the cutoff 17 mm and FAC with the cutoff 35% revealed non-significant hazard ratios (HR) of 0.75 (95%CI 0.396–1.421, *p* = 0.38) and 0.845 (95%CI 0.383–1.867, *p* = 0.68), respectively. TAPSE with the cutoff 19 mm and visual eyeballing significantly predicted survival with HRs of 0.512 (95%CI 0.296–0.886, *p* = 0.017) and 1.631 (95%CI 1.101–2.416, *p* = 0.015), respectively. Conclusions: This large-scale all-comer study confirms that RVF is one of the main drivers of mortality in patients with severe isolated TR. However, the current cut-offs for established echocardiographic parameters did not predict survival. Further studies should investigate the prognostic value of higher thresholds for RVF parameters in these patients.

## 1. Background

Evaluation and gradation of right ventricular systolic function (RVF) is an integral part of every transthoracic echocardiographic examination (TTE) [1,2,3]. Current recommendations propose numerous parameters that can be measured, only few of these are actually applied in daily clinical practice, namely tricuspid annular plane systolic excursion (TAPSE), tissue Doppler velocity of the free lateral wall (S’), fractional area change (FAC), and visual quantification also referred to as “eyeballing” [4]. A lower TAPSE value is associated with lower cardiac index and worse survival [5,6].

Presence of high trans-valvular pendulous volume leads to backward-unloading of the ventricle and complicates the functional assessment of the affected ventricle. Established cut-offs for normal systolic performance can overestimate true systolic function, as it has previously been described for left ventricular ejection fraction (LVEF) in the setting of severe mitral regurgitation [7]. Therefore, a similar mechanism may be assumed for RVF in the presence of severe tricuspid regurgitation (TR).

Tricuspid regurgitation is a common entity and often occurs as a bystander of other severe cardiac diseases. It is frequently associated with mitral regurgitation, aortic stenosis, and systolic left heart failure, all of them significant drivers of mortality [8,9]. Therefore, to truly understand the clinical and prognostic relevance of RV function parameters, a cohort needs to be analyzed where there is “isolated” TR, with no previous heart surgery or intervention, no other relevant valvular lesion, and normal systolic left ventricular function.

The present study sought to evaluate the prognostic value of established cutoffs for several parameters frequently applied to grade RVF in the presence of isolated severe TR.

## 2. Methods

A retrospective analysis was performed entailing all patients who underwent transthoracic echocardiography at our institution between 1 January 2013 and 31 December 2016. Survival was retrieved for 31 December 2018 from the national death registry. The study protocol conformed to the ethical guidelines of the Declaration of Helsinki and was approved by the local ethics committee (EK #1179/2019).

### 2.1. Study Population

The study population consisted of consecutive patients who were graded with TR greater than moderate. All patients with impaired left ventricular systolic function (LVEF < 50%), previous cardiac surgery, congenital heart disease, or any concomitant valvular defect with a severity greater than mild to moderate, as well as repeat examinations were excluded. Subsequently, the remaining patients were classified according to TR vena contracta width (VC). Only patients with severe TR (defined as VC ≥ 7 mm) were included into the final analysis.

### 2.2. Echocardiographic Assessment

Echocardiograms were performed using commercially available equipment (Vivid E9, Vivid7, GE Healthcare, Chicago, IL, USA) according to a standardized protocol by physicians and sonographers blinded to clinical data and outcomes. For this study, all stored loops were re-read by one experienced examiner (AK). Cardiac chamber size and function were assessed according to the recommendations of the European Association of Cardiovascular Imaging using 2D images [2].

### 2.3. Right Heart Dimensions

The maximal basal RV diameter was measured in an RV focused apical four-chamber view [2]. Size of the right atrium was assessed by the major dimension measured in the apical four-chamber view [10].

### 2.4. Tricuspid Regurgitation

Comprehensive assessment of TR was performed using vena contracta width (VC), effective regurgitant orifice area (EROA), and regurgitant volume (RegV) using the proximal isovelocity surface area (PISA) method [11].

### 2.5. Right Ventricular Function

#### 2.5.1. Visual “Eyeballing”

Visual qualitative quantification of RVF is the most commonly applied parameter in daily clinical practice [4]. Due to the anatomy of the RV, several echocardiographic views need to be included in the assessment [1,3]. “Eyeballing” can reliably detect severely reduced RVF and differentiate between normal and reduced RVF [12]. As supplemental material, two video loops are provided, one with normal RVF (Supplemental Material 1) and one with reduced RVF (Supplemental Material 2).

#### 2.5.2. TAPSE

In an apical 4-chamber view the maximal systolic excursion of the lateral tricuspid annulus was measured by M-Mode. A TAPSE of <17 mm indicates RV dysfunction [3].

#### 2.5.3. Tissue Doppler Velocity of the Free Lateral Wall (S’)

This parameter provides the longitudinal velocity of the tricuspid annular plane by tissue Doppler imaging. The sample volume was placed in the basal segment of the free lateral wall of the right ventricle. An S’ value of 9.5 cm/s indicates RV dysfunction [2,3].

#### 2.5.4. Fractional Area Change (FAC)

The endocardial RV boarders were traced during end-diastole (ED) and end-systole (ES) and the respective area was measured. The following formula was applied: 100× (RV-Area ED—RV-Area ES)/RV-Area ED. A value of less than 35% indicates RV systolic dysfunction [3].

#### 2.5.5. Global Longitudinal Strain

Global strain of the free lateral wall of the right ventricle (GLS-RVFW) was calculated by averaging peak systolic strain of the three segments (basal, mid, apical) of the free lateral wall in an RV focused 4-chamber-view. A free-wall-strain of less than −20% indicates reduced RVF [3].

### 2.6. Clinical Risk Factors and Laboratory Measurements

A systematic exploration of the centralized patient management system of Vienna revealed age, sex, and traditional cardiovascular risk factors according to the respective guidelines as previously described [13]. Venous blood samples were analyzed according to the local laboratory standard procedures.

### 2.7. Clinical Follow-Up and Study Endpoints

Mortality was analyzed by a retrieval query of the Austrian Death Registry. Record linkage indicated date of death and cause of death encoded according to the International Code of Diseases, Ver. 10 (ICD10). Austrian law demands that all deaths of Austrian citizens (also in foreign countries, if reported to Austrian officials) have to be recorded in the central Austrian death registry, which allows an almost complete follow up of all patients [14].

### 2.8. Statistical Analysis

Continuous variables were tested for normal and non-normal distribution by Shapiro-Wilk test. Groups were compared with the *t*-test (normal distribution) or with the Mann–Whitney *U* test (non-normal distribution). Continuous variables are given as median and interquartile range (Q1–Q3) in the presence of non-normal distribution and with mean ± standard deviation in the presence of normal distribution. Categorical parameters are presented as absolute numbers and percentages.

Cox proportional hazard regression analysis was applied to assess the effect of RVF on 2-year survival. For TAPSE, S’, FAC, and GLS-RVFW the cutoffs recommended by the current guidelines were applied. To explore possibly better thresholds, a second calculation was performed for each parameter, using the parameter’s median value in this cohort as a cutoff. To account for potential confounding effects, we formed a confounder cluster encompassing age, female sex, creatinine, RA and RV size, and maximal TR velocity (continuous variable).

Intraclass correlation coefficient was calculated for 20 randomly selected patients regarding the analyzed RV function parameters.

Two-sided *p*-values < 0.05 were used to indicate statistical significance. SPSS Version 24 (IBM SPSS, Chicago, IL, USA) was used for all analyses.

## 3. Results

### 3.1. Patient Selection

A total of 36,000 patients received transthoracic echocardiography at our institution between 1 January 2013 and 31 December 2016, 590/36,000 (1.6%) fulfilled the inclusion criteria of severe isolated tricuspid regurgitation (initial diagnosis in the report) with normal systolic left ventricular function and no other valvular pathology graded as more than mild-to-moderate. We excluded 328 patients due to insufficient image quality, measurements were performed on the remaining 262 patients. Subsequently, 42 patients were excluded due to less than severe TR indicated by a VC < 7 mm (Supplemental Material 3).

### 3.2. Baseline Characteristics

The final cohort consisted of 220 patients, 88/220 (40%) were male. Median age was 69 years (IQR 52–79), median follow-up was 34.5 (18.5–53) months, all-cause two-year mortality was 29% (survival data were available for all included patients). Cardiovascular comorbidities were coronary artery disease in 28%, diabetes mellitus in 20%, and arterial hypertension in 59% (Table 1). Additional comorbidities were COPD in 34/220 (15%), a history of pulmonary embolism in 18/220 (8%), systemic lupus erythematodes in 4/220 (2%), a malignant disease (Mamma carcinoma, renal cell carcinoma, Hodgkin lymphoma, Non-Hodgkin lymphoma, Insulinoma, prostate cancer, mantle cell lymphoma, multiple myeloma, polycythemia vera) in 14/220 (6%), and 3/220 (1%) patients with lung fibrosis.

### 3.3. Tricuspid Regurgitation

Tricuspid regurgitation was severe in all patients defined by a VC ≥ 7 mm. Median VC was 10 mm (8–12), median EROA was 0.4 cm^2^ (0.25–0.54), and median regurgitant volume was 44 mL (34–60) (Table 1).

### 3.4. Right Ventricular Function and 2-Year Survival

The parameter TAPSE was available in 192/220 (87%) of the patients. Median TAPSE was 19 mm (15–22). It was significantly higher in those who survived beyond two years compared to the non-survivors (19 mm vs. 17 mm, *p* = 0.034). However, the TAPSE-cutoff of 17 mm did not differentiate between these groups (*p* = 0.137).

**Figure 1:**
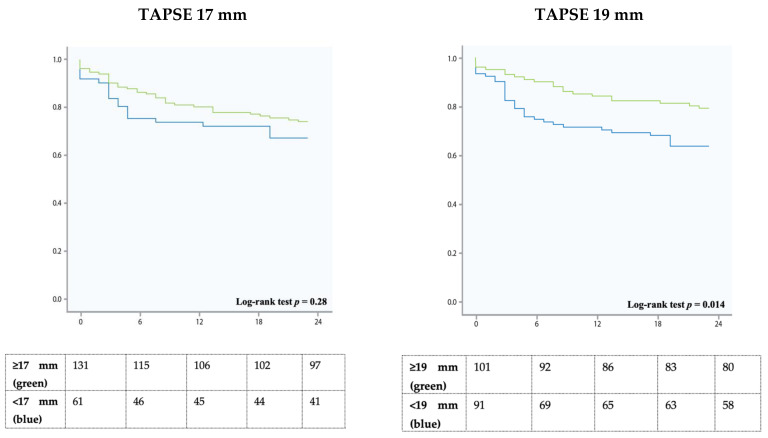
Kaplan Meier survival analysis showing 2-year survival of patients with tricuspid annular plane systolic excursion (TAPSE) cutoffs 17 mm and 19 mm (right). The green line indicates higher values (≥17 mm on the left, ≥19 mm on the right), the blue line indicates lower values (<17 mm on the left, <19 mm on the right).

For the cutoff 17 mm, multivariate analysis revealed a hazard ratio (HR) of 0.75 (95%CI 0.396–1.421, *p* = 0.38). For the cutoff 19 mm, multivariate analysis revealed a HR of 0.526 (95%CI 0.286–0.968, *p* = 0.039) (Figure 1, Table 2).

The parameter FAC was available in 194/220 (88%) of the patients. Median FAC was 42% (30–52). There was no statistically significant difference between the two groups (44% vs. 40%, *p* = 0.13). The FAC-cutoff of 35% did not differentiate between the two groups (*p* = 0.56).

**Figure 2:**
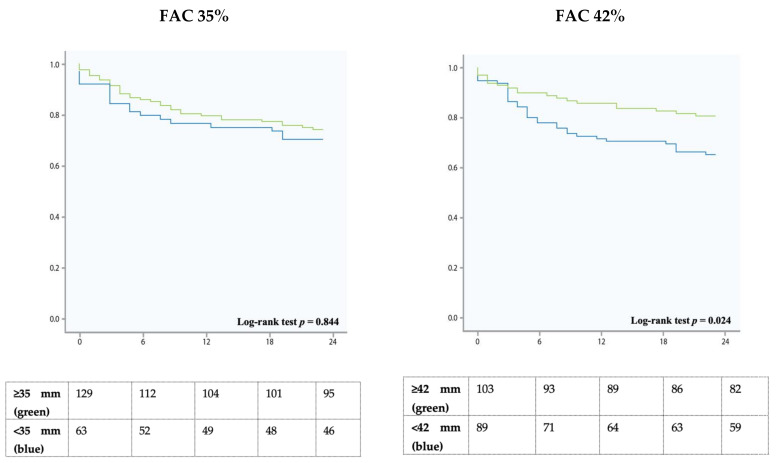
Kaplan Meier survival analysis showing 2-year survival of patients with the fractional area change (FAC) cutoffs 35% and 42% (right). The green line indicates higher values (≥35% on the left, ≥42% on the right), the blue line indicates lower values (<35% on the left, <42% on the right).

For the cutoff 35%, multivariate analysis revealed a HR of 0.845 (95%CI 0.383–1.867, *p* = 0.68). For the cutoff 42%, multivariate analysis revealed a HR of 0.495 (95%CI 0.222–1.103, *p* = 0.08) (Figure 2, Table 2).

The parameter S’ was available in 153/220 (69%) of the patients. Median S’ was 11 cm/s (8.5–12). There was no statistically significant difference between the two groups (11 cm/s vs. 9 cm/s, *p* = 0.37).

For the cutoff 9.5 cm/s, multivariate analysis revealed a HR of 1.053 (95%CI 0.516–2.15, *p* = 0.89). For the cutoff 11 cm/s, multivariate analysis revealed a HR of 1.048 (95%CI 0.520–2.112, *p* = 0.89) (Table 2).

The parameter GLS-RVFW was available in 135/220 (61%) of the patients. Median GLS-RVFW was −20% (−11–(−27)). There was no statistically significant difference between the two groups (−21% vs. −17%, *p* = 0.48). For the cutoff −20%, multivariate analysis revealed a HR of 0.338 (95%CI 0.113–1.008, *p* = 0.052) (Table 2).

Visual “eyeballing” of RVF was performed in all patients. A total of 111/220 (50.5%) of the patients were graded as “reduced function”. There were significantly more patients with reduced RVF in the non-survivors when compared to the survivors (65% vs. 45%, *p* = 0.005). Visual gradation into “normal RVF” and “reduced RVF” showed a significant association with two year survival in multivariate analysis (HR 1.631, 95%CI 1.101–2.416, *p* = 0.015) (Table 2).

In all analyses, age and creatinine were significantly associated with mortality (Table 2).

### 3.5. Interrater Variability

Interrater variability was tested for 20 randomly selected patients by intraclass correlation coefficient. This was 0.94 (95%CI 0.83–0.98, *p* < 0.001) for visual gradation of RV function, 0.92 (95%CI 0.74–0.97, *p* < 0.001) for TAPSE, 0.99 (95%CI 0.95–1.0, *p* < 0.001) for S’, 0.87 (95%CI 0.61–0.96, *p* < 0.001) for FAC, and 0.9 (95%CI 0.61–0.98, *p* = 0.001) for GLS-RVFW.

## 4. Discussion

This large-scale all-comer study confirms the prognostic importance of RVF in patients with severe TR. However, our data challenges the current cutoffs determined by the guidelines. None of the parameters predicted survival if the established cut-offs were applied. A higher TAPSE threshold for lower limit of normality (TAPSE 19 mm) showed a significantly better risk discrimination for all-cause two-year survival.

Prevalence of isolated severe TR is extremely low. Over the four-years of investigation, of the screened 36,000 patients only 590/36,000 (1.6%) had isolated severe TR. Patients were relatively young with a median age of 69 (IQR 52–79), and prognosis was unfavorable with a 2-year mortality of 29%.

Numerous studies have shown that reduced RVF is associated with worse prognosis in severe TR [15,16]. However, it still remains unclear if the same lower limits of normality apply for each of the parameters as in patients without TR.

Severe pendulous volume caused by atrio-ventricular valvular regurgitation leads to hyperdynamic ventricular function in order to maintain sufficient stroke volume. This concept is the reason why an LVEF below 60% is understood to indicate incipient LV dysfunction and therefore is an indication for mitral valve surgery in severe mitral regurgitation [7]. In the setting of severe TR, the current guidelines do not provide similarly adapted cutoffs for functional parameters, e.g., for TAPSE, S’, and FAC.

Nevertheless, it can be assumed that in severe TR a higher than usual longitudinal and radial function is needed to maintain stroke volume. Our data provides new insights into this matter and challenges the current cutoffs in the presence of severe TR. Neither in univariate nor in multivariable analysis these cutoffs predicted survival. However, when applying higher cutoffs (19 mm in TAPSE), patients with the higher values showed significantly better survival. The same applied for FAC, S’, and GLS-RVFW, however without statistical significance.

The presented data were collected from a small number of patients in a single center and may reflect referral bias. The findings therefore indicate that the guideline cutoffs are less reflective of risk in this particular cohort. Consequently, the results can only be hypothesis generating. Future studies should prospectively investigate higher cut-offs for TAPSE, GLS-RVFW, S’, FAC, and RVEF in patients with severe TR.

Interestingly, visual estimation of RVF (“eyeballing”) was able to predict survival in multivariate analysis. This adds to the discussion regarding the optimal parameter for gradation of RVF. TAPSE, GLS-RVFW, and S’ depict longitudinal function of the free lateral wall of the RV, and FAC and RVEF (derived by 3D echocardiography or cardiac magnetic resonance imaging) depict volumetric changes during the cardiac cycle. Visual “eyeballing” by the experienced examiner might be able to give a more holistic estimation of RVF incorporating both longitudinal and volumetric function. This hypothesis is supported by previous data from our group that could show good diagnostic accuracy by eyeballing regarding the differentiation into “normal” and “reduced” RVF [12].

As the right ventricle quickly adapts to changes in volume and pressure, when evaluating patients with severe TR it is important to take into consideration not only parameters of RVF but also pulmonary pressures and right heart dimensions [17]. Recently, a new scheme of patterns for patients with severe TR was proposed by Dietz et al. incorporating both RV size and RV (dys)function. Here, RV dysfunction was a predictor for worse prognosis [18]. The authors applied a TAPSE <17 to characterize patients with reduced RVF. A higher cut-off might produce even more pronounced results.

In the analyses performed for this study, the confounders age, RV size, and creatinine contributed significantly to two-year all-cause mortality. Right ventricular size was characterized by basal diameter by our group. Future studies should focus on more comprehensive parameters, particularly indexed 3D end-diastolic RV volume which can be derived in most patients if hardware and software are available [19]. In addition, particular attention should be paid towards associated organ damage, such as renal and liver dysfunction [20].

### 4.1. Limitations

Due to the retrospective design of this study, measurements could only be performed on the available images. This lead to a high drop-out rate due to suboptimal image quality.

The investigated parameters were not available for all patients (e.g., TAPSE *n* = 192/220 (87%), GLS-RVFW *n* = 135/220 (61%)). This may explain why differences between the lower and higher threshold in S’ and GLS-RVFW did not reach statistical significance. Future studies should investigate these parameters prospectively and in larger cohorts.

To correct for potential bias on survival, all significant left-heart valvular lesions (graded more than mild-to-moderate) and systolic dysfunction (LV systolic function graded not normal) were excluded. However, heart failure with preserved ejection fraction (HFpEF) could not be excluded. At the same time, as only patients with isolated TR were analyzed, the reported findings may not be generalized for all patients with TR as most will present with additional left heart disease.

TR Vmax measurement was important in this analysis for the assessment of pulmonary pressures. However, in the presence of severe TR, TR Vmax can be false-low despite high pulmonary pressures due to pressure equalization between the RA and the RV. A triangular shaped CW Doppler signal in combination with a low TR Vmax signal is a sign for the presence of pressure equalization. This was present in 13/220 (6%) patients in this cohort.

As for mechanism of tricuspid regurgitation, the analyzed patients had functional (secondary) TR, however, there were patients with cardiac implantable electronic devices (CIED). In our clinical experience it is oftentimes not possible to exclude CIED induced TR from retrospective datasets. Therefore, unrecognized patients with primary or structural TR may have been included in the presented data.

As the analysis is based on a single center analysis, the results may not generalize to other institutions and there is concomitant referral bias that can influence who receives TTE and mortality rates.

### 4.2. Conclusions

This large-scale all-comer study confirms that RVF is one of the main drivers of mortality in patients with severe isolated TR. However, the current cut-offs for established echocardiographic parameters did not predict survival. Further studies should investigate the prognostic value of higher thresholds for RVF parameters in these patients.

## Figures and Tables

**Table 1:** Baseline characteristics.

	Total	2-Year Survivors	2-Year Non-Survivors	*p*-Value
Number of patients, *n* (%)	220 (100)	157 (71)	63 (29)	
Age, years (IQR)	69 (52–79)	66 (50–76)	77 (63–82)	**<0.001**
Female sex, *n* (IQR)	132 (60)	99 (63)	33 (52)	0.132
Creatinine, mg/dL (IQR)	0.97 (0.8–1.3)	0.93 (0.77–1.15)	1.2 (0.9–1.6)	**<0.001**
NYHA class, *n* (%)				
I, *n* (IQR) II, *n* (IQR) III, *n* (IQR) IV, *n* (IQR)	122 (56) 24 (11) 33 (15) 9 (4)	93 (60) 17 (11) 24 (16) 6 (4)	29 (45) 7 (11) 9 (14) 3 (5)	0.40
NT-proBNP, pg/mL (IQR)	1910 (856–3815)	1446 (665–2718)	3146 (1914–5673)	**<0.001**
**Cardiovascular comorbidities**				
CAD, *n* (%)	61 (28)	42 (27)	19 (30)	0.83
DM, *n* (%)	43 (20)	32 (20)	11 (18)	0.45
HTN, *n* (%)	129 (59)	87 (55)	42 (67)	0.27
Atrial fibrillation, *n* (%)	107 (49)	76 (48)	31 (49)	0.70
**Cardiac dimensions**				
RV size, mm (±SD)	45 (±9.4)	45 (±9.6)	47 (±8.7)	0.17
RA size, mm (± SD)	68 (±9.7)	68 (±9.5)	68 (±10.5)	0.87
LV size, mm (±SD)	39 (±7)	39 (±7)	38 (±6.8)	0.44
LA size, mm (IQR)	60 (55–68)	60 (55–68)	60 (54–69)	0.8
**Pulmonary hypertension**				
TR Vmax, m/s (IQR)	3.7 (3.1–4.5)	3.6 (3–4.4)	4 (3.2–4.6)	0.26
sPAP, mmHg (IQR)	70 (53–96)	65 (51–95)	77 (59–100)	0.16
**Tricuspid regurgitation**				
VC, mm (IQR)	10 (8–12)	9.5 (8–11)	11 (8–12)	**0.049**
EROA, cm^2^ (IQR)	0.4 (0.25–0.54)	0.4 (0.28–0.55)	0.37 (0.21–0.54)	0.14
RegV, mL (IQR)	44 (34–60)	45 (35–63)	43 (29–58)	0.07
**Right ventricular function**				
TAPSE, mm (IQR)	19 (15–22)	19 (16–23)	17 (14–21)	**0.048**
FAC, % (IQR)	42 (30–52)	44 (30–52)	40 (28–50)	0.12
S’, cm/s (IQR)	11 (8.5–12)	11 (9–13)	9 (8–12)	0.72
GLS-RVFW, % (IQR)	−20 (−11–(−27))	−21 (−11–(−28))	−17 (−12–(−26))	0.49
RVF reduced (visual assessment), *n* (%)	111 (50.5)	70 (45)	41 (65)	**0.035**
FAC ≥ 35%, *n* (%)	129 (58.6)	96 (61)	33 (52)	0.93
TAPSE ≥ 17 mm, *n* (%)	131 (59.5)	100 (64)	31 (49)	0.33

NYHA = New York Heart association. CAD = coronary artery disease. DM = diabetes mellitus. HTN = arterial hypertension. RV = right ventricle. RA = right atrium. LV = left ventricle. LA = left atrium. TR V max = tricuspid regurgitation maximal velocity. sPAP = systolic pulmonary artery pressure. VC = vena contracta width. EROA = effective regurgitant orifice. RegV = regurgitant volume. TAPSE = tricuspid annular plane systolic excursion. FAC = fractional area change. S’ = Tissue Doppler velocity of the free lateral wall. GLS-RVFW = global longitudinal strain of the right ventricular free wall. RVF = right ventricular function. IQR = interquartile range. SD = standard deviation. Bold numbers indicate statistical significance.

**Table 2:** Cox proportional hazard regression analysis to assess the effect of different RVF parameters on survival during 2-year follow-up.

TAPSE (Cutoff 17 mm)	HR	95% CI		*p*-Value	HR	95% CI		*p*-Value
Univariate Analysis					Multivariate Analysis			
Increasing Age	1.033	1.016	1.051	**<0.001**	1.043	1.021	1.067	**<0.001**
Female sex	1.064	0.648	1.748	0.81	0.881	0.459	1.69	0.703
Creatinine	1.632	1.358	1.96	**<0.001**	2.175	1.511	3.13	**<0.001**
TR-Vmax (continuous)	1.144	0.876	1.493	0.32	1.268	0.888	1.81	0.19
RV diameter	1.017	0.991	1.044	0.19	1.046	1.006	1.089	**0.025**
RA diameter	1.0	0.975	1.026	0.97	0.974	0.94	1.01	0.15
TAPSE ≥ 17 mm	0.741	0.426	1.287	0.29	0.75	0.396	1.421	0.38
**TAPSE (cutoff 19 mm)**	**HR**	**95% CI**		***p*-value**	**HR**	**95% CI**		***p*-value**
**Univariate analysis**					**Multivariate analysis**			
Increasing Age	1.033	1.016	1.051	**<0.001**	1.041	1.019	1.063	**<0.001**
Female sex	1.064	0.648	1.748	0.81	0.842	0.442	1.607	0.6
Creatinine	1.632	1.358	1.96	**<0.001**	2.131	1.48	3.068	**<0.001**
TR-Vmax (continuous)	1.144	0.876	1.493	0.32	1.225	0.865	1.736	0.25
RV diameter	1.017	0.991	1.044	0.19	1.045	1.004	1.087	**0.029**
RA diameter	1.0	0.975	1.026	0.97	0.978	0.944	1.012	0.20
TAPSE ≥19 mm	0.512	0.296	0.886	**0.017**	0.526	0.286	0.968	**0.039**
**FAC (cutoff 35%)**								
**Univariate analysis**					**Multivariate analysis**			
Increasing Age	1.033	1.016	1.051	**<0.001**	1.05	1.026	1.076	**<0.001**
Female sex	1.064	0.648	1.748	0.81	0.724	0.369	1.42	0.35
Creatinine	1.632	1.358	1.96	**<0.001**	2.49	1.725	3.594	**<0.001**
TR-Vmax (continuous)	1.144	0.876	1.493	0.32	1.37	0.936	2.0	0.11
RV diameter	1.017	0.991	1.044	0.19	1.049	1.004	1.097	**0.032**
RA diameter	1.0	0.975	1.026	0.97	0.974	0.94	1.008	0.13
FAC ≥35%	0.944	0.527	1.689	0.85	0.845	0.383	1.867	0.68
**FAC (cutoff 42%)**								
**Univariate analysis**					**Multivariate analysis**			
Increasing Age	1.033	1.016	1.051	**<0.001**	1.054	1.028	1.08	**<0.001**
Female sex	1.064	0.648	1.748	0.81	0.737	0.372	1.458	0.38
Creatinine	1.632	1.358	1.96	**<0.001**	2.336	1.62	3.367	**<0.001**
TR-Vmax (continuous)	1.144	0.876	1.493	0.32	1.208	0.813	1.793	0.35
RV diameter	1.017	0.991	1.044	0.19	1.041	0.997	1.087	0.07
RA diameter	1.0	0.975	1.026	0.97	0.974	0.94	1.01	0.15
FAC ≥42%	0.528	0.299	0.931	**0.027**	0.495	0.222	1.103	0.08
**Eyeballing**								
**Univariate analysis**					**Multivariate analysis**			
Increasing Age	1.033	1.016	1.051	**<0.001**	1.051	1.027	1.075	**<0.001**
Female sex	1.064	0.648	1.748	0.81	0.858	0.454	1.619	0.64
Creatinine	1.632	1.358	1.96	**<0.001**	2.043	1.424	2.931	**<0.001**
TR-Vmax (continuous)	1.144	0.876	1.493	0.32	1.143	0.78	1.673	0.49
RV diameter	1.017	0.991	1.044	0.19	1.026	0.983	1.071	0.24
RA diameter	1.0	0.975	1.026	0.97	0.97	0.936	1.005	0.09
Eyeballing	1.341	1.045	1.722	**0.021**	1.631	1.101	2.416	**0.015**

TAPSE = tricuspid annular plane systolic excursion. TR Vmax = tricuspid regurgitation maximal velocity. RV = right ventricle. RA = right atrium. FAC = fractional area change. HR = hazard ratio. CI = confidence interval. Bold numbers indicate statistical significance.

## Supplementary Materials

The following are available online at https://www.mdpi.com/article/10.3390/jcm10112266/s1, Video S1: normal right ventricular function, Video S2: reduced right ventricular function, Figure S1: STROBE algorithm showing patient selection.
