# Supplementary material for: Prognostic Value of Echocardiographic Right Ventricular Function Parameters in the Presence of Severe Tricuspid Regurgitation
Source: J Clin Med. 2021 May 24;10(11):2266. doi: 10.3390/jcm10112266 (PMC8197252; doi:10.3390/jcm10112266)
Supplement: Supplementary file 1 [file jcm-10-02266-s001.zip › supplemental material 3.pdf]

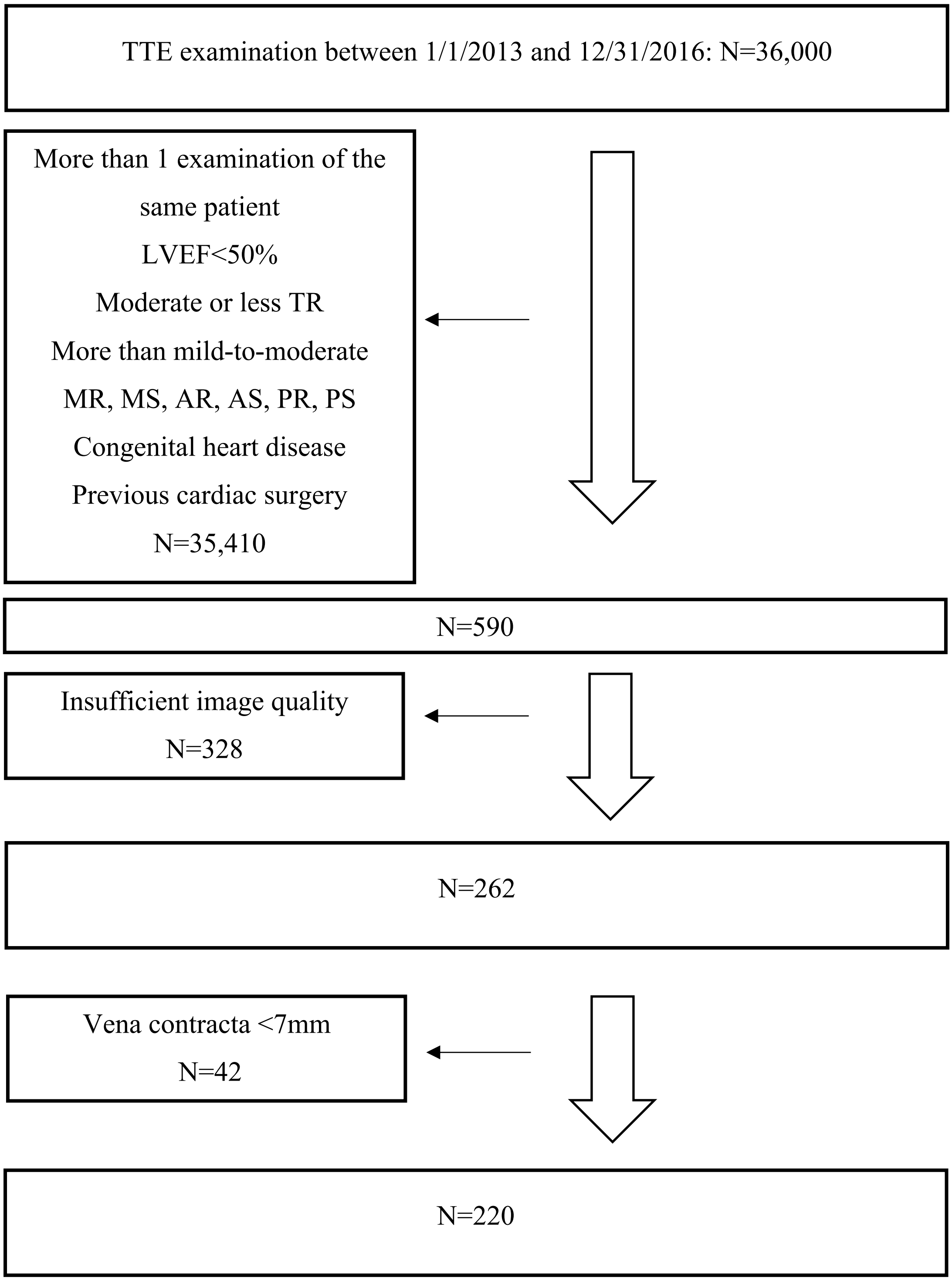


Supplementary material 3: STROBE algorithm showing patient selection. TTE = transthoracic echocardiography, LVEF = left ventricular ejection fraction, MR = mitral regurgitation, MS = mitral stenosis, AR = aortic regurgitation, AS = aortic stenosis, PR = pulmonary regurgitation, PS = pulmonary stenosis.
